# Clinical factors associated with the recovery of cardiovascular autonomic neuropathy in patients with type 2 diabetes mellitus

**DOI:** 10.1186/s12933-019-0830-4

**Published:** 2019-03-11

**Authors:** Ji Eun Jun, Seung-Eun Lee, Min Sun Choi, Sung Woon Park, You-Cheol Hwang, Jae Hyeon Kim

**Affiliations:** 1 Division of Endocrinology and Metabolism, Department of Internal Medicine, Samsung Medical Center, Sungkyunkwan University School of Medicine, 81 Irwon-Ro, Gangnam-Gu, Seoul, 135-710 Republic of Korea; 2 Department of Endocrinology and Metabolism, Kyung Hee University Hospital at Gangdong, Kyung Hee University School of Medicine, Seoul, Republic of Korea; 3 Department of Clinical Research Design & Evaluation, SAIHST, Sungkyunkwan University, 81 Irwon-Ro, Gangnam-Gu, Seoul, Republic of Korea

**Keywords:** Cardiovascular autonomic neuropathy, Recovery, Type 2 diabetes mellitus

## Abstract

**Background:**

Cardiovascular autonomic neuropathy (CAN) is a major cause of morbidity and mortality in diabetes patients. Although several risk factors for CAN progression have been established, whether CAN is reversible remains unclear and the clinical factors associated with CAN recovery have not been identified. This study aimed to determine clinical factors related to CAN recovery.

**Methods:**

Type 2 diabetes patients with CAN but free of cardiovascular disease at baseline were enrolled and followed for 2–3 years in this retrospective longitudinal study. CAN was classified as early (one abnormal parasympathetic test), definite (two or more abnormal parasympathetic tests), severe (definite plus orthostatic hypotension), or atypical (early plus orthostatic hypotension or orthostatic hypotension alone) based on Ewing’s method. CAN recovery was classified as partial or complete: Partial recovery was defined as one-step improvement in CAN stage (early to normal, definite to early, or severe to definite), including the disappearance of only one abnormal result in any stage. Complete recovery was defined as normalization from definite or severe CAN.

**Results:**

Among 759 subjects with CAN, 29.9% (n = 227) experienced CAN recovery, and 1.2% (n = 9) recovered completely. In a multivariate model, younger age (odds ratio [OR] per 5-year decrease 1.49; 95% confidence interval [CI] 1.25–1.78, *P* < 0.001), shorter duration of diabetes (OR per 5-year decrease 1.33; 95% CI 1.05–1.67, *P* = 0.016), presence of micro/macroalbuminuria (OR 0.34; 95% CI 0.15–0.78, *P* = 0.011), body weight reduction (OR per 1-kg decrease 1.11; 95% CI 1.02–1.21, *P* = 0.016), and HbA1c reduction (OR per 1% decrease 1.32; 95% CI 1.05–1.67, *P* = 0.019) were significantly associated with composite events of partial and complete CAN recovery. Age had the highest relative significance among the associated clinical factors. In addition, younger age was the only significant factor in complete CAN recovery.

**Conclusions:**

Younger age was the most important factor in CAN recovery in subjects with type 2 diabetes, including recovery from the definite or severe stage. HbA1c reduction, body weight reduction, no concurrent micro/macroalbuminuria, and shorter duration of diabetes were also significantly associated with CAN recovery.

**Electronic supplementary material:**

The online version of this article (10.1186/s12933-019-0830-4) contains supplementary material, which is available to authorized users.

## Background

Cardiovascular autonomic neuropathy (CAN) is a major cause of morbidity and mortality in patients with diabetes [1]. CAN results from damage to the autonomic nerve fibers that innervate the heart and blood vessels and eventually leads to abnormalities in cardiovascular dynamics [2] and anatomy [3]. Diabetic autonomic neuropathy originates from complex interactions among glycemic control, glycemic fluctuation, diabetes duration, age-related neuronal deficits, and other cardiovascular risk factors such as hypertension, hyperlipidemia, obesity, and smoking status [4–7]. Other diabetic microvascular complications have also been identified as clinical predictors of CAN [8].

The time course of changes in CAN and the development of early cardiac functional abnormalities from each CAN stage are poorly understood [9]. It is estimated that subclinical CAN, which presents as impaired heart rate variability (HRV) due to parasympathetic denervation, will progress to clinical CAN, which results from sympathetic augmentation or denervation, within 5 years [9]. One prospective study demonstrated that during 2 years of follow-up, most HRV indices deteriorated gradually in all patients with type 1 or type 2 diabetes who had CAN but not overt cardiovascular disease (CVD) [10].

Although several risk factors for CAN progression have been established, whether CAN is reversible over time remains unclear, and characteristics associated with CAN recovery have not been identified. In one study, serum adiponectin was associated with favorable changes in HRV [11], while increases in HbA1c and body mass index (BMI) over time were associated with aggravation of HRV indices in patients with type 2 diabetes [12]. CAN has been considered an irreversible complication [13], but patients with type 1 diabetes did recover from early-stage CAN as rapidly as 1 year after the initiation of strict glycemic control [14]. However, glycemic control has a larger effect on the prevention of diabetic neuropathy in type 1 diabetes compared to type 2 diabetes, suggesting that the underlying mechanism might be different [13]. This study thus seeks to evaluate the reversibility of CAN and determine clinical factors related to CAN recovery in patients with type 2 diabetes.

## Methods

### Study subjects

**Fig. 1:**
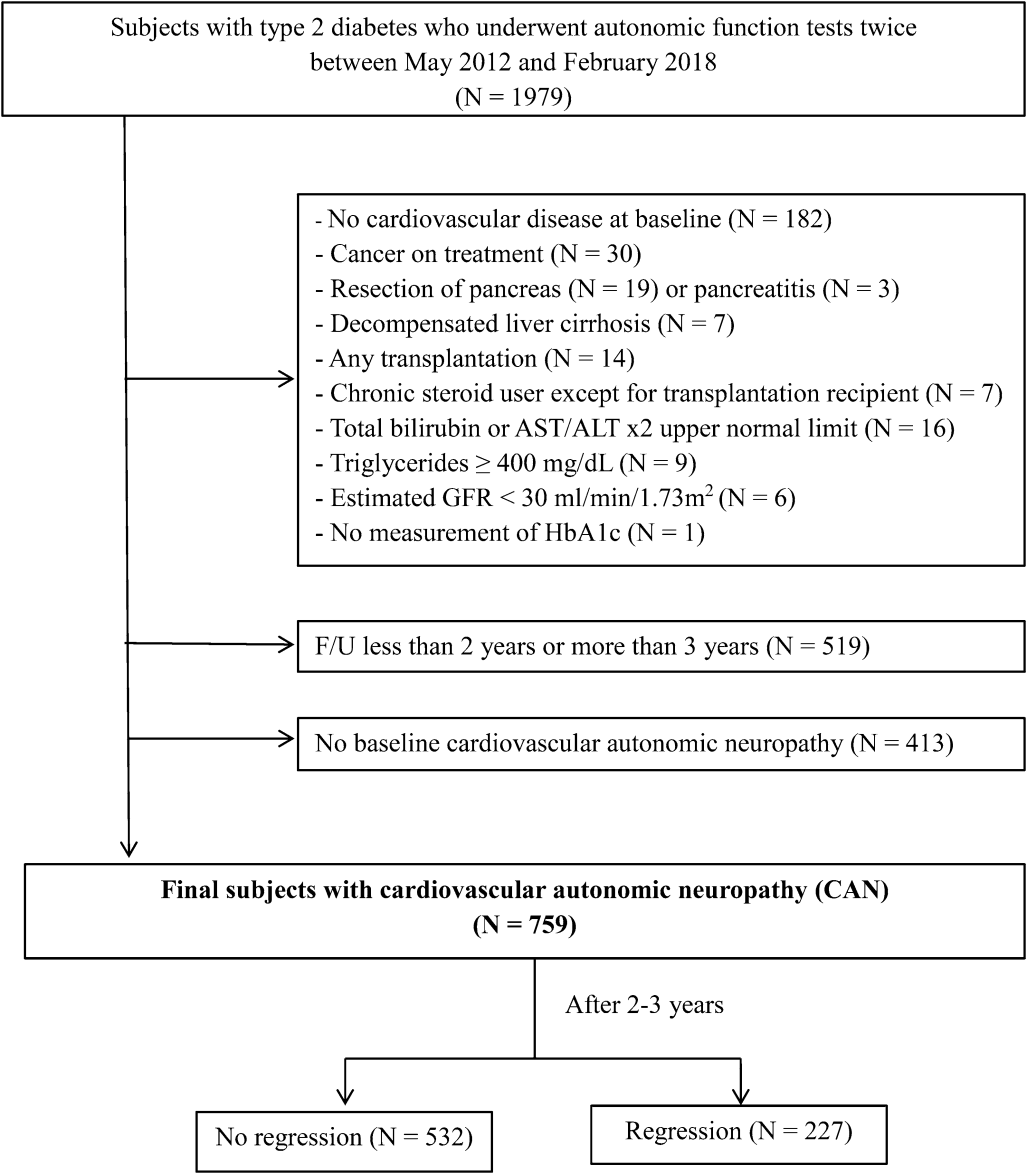
Inclusion and exclusion criteria for the study subjects. AST, aspartate transaminase; ALT, alanine aminotransferase; GFR, glomerular filtration rate

This retrospective, longitudinal study included adult patients (≥ 20 years) with type 2 diabetes who underwent cardiovascular autonomic reflex tests (CARTs) twice during a 2–3-year period at Samsung Medical Center in Korea. Between May 2012 and February 2018, a total of 1979 subjects were sequentially screened for eligibility (Fig. 1). Among them, subjects with a history of CVD (n = 182), cancer (n = 30), resection of the pancreas (n = 19), pancreatitis (n = 3), decompensated liver cirrhosis (n = 7), transplantation of any organ (n = 14), chronic steroid use (n = 7), a level of total bilirubin or liver enzymes more than 2 times higher than the upper normal limit (n = 16), triglycerides (TG) ≥ 400 mg/dL (n = 9), an estimated glomerular filtration rate (eGFR) < 30 ml/min/1.73 m^2^ (n = 6), or missing HbA1c measurements (n = 1) were excluded. In addition, those who were followed for less than 2 years or more than 3 years (n = 519), and those who did not have CAN at baseline (n = 413) were also excluded. In the end, 759 patients were included in this study, which was approved by the Ethics Committee of Samsung Medical Center and performed according to the Declaration of Helsinki (IRB Number: 2018-09-066).

### CAN assessments

CAN assessments were based on Ewing’s method, which includes a battery of four non-invasive CARTs [15]: HRV during deep breathing (exhalation: inhalation [E:I] ratio), while standing (30:15 ratio), and during the Valsalva maneuver (Valsalva ratio) as measures of parasympathetic function, and blood pressure (BP) response to standing as a measure of sympathetic function. HRV was assessed automatically from electrocardiography recordings using the DICAN system (Medicore Co., Ltd., Seoul, Korea). Patients were advised to avoid strenuous physical exercise, tobacco, and alcohol in the 24 h preceding the test and to avoid coffee and food for at least 3 h prior to the test. Medications such as antihistamines, antidepressants, acetaminophen, and β-blockers were withheld for 12 h prior to the test.

Each of the three parasympathetic function tests described above was classified as normal or abnormal according to age-specific reference ranges [16–18] and postural BP changes (decreases in systolic BP) ≥ 30 mmHg were considered abnormal. Postural BP changes were graded as follows: normal, 0; each borderline test, 0.5; and each abnormal test, 1. Parasympathetic function tests were graded as: normal, 0; each abnormal test, 1; (Additional file 1: Table S1). Total CAN score was the sum of the partial points obtained from each of the four autonomic function tests [19].

CAN staging was performed as follows [15]: (1) patients whose results were all within normal range were classified as normal; (2) one abnormal result among the three parasympathetic tests was defined as early CAN; (3) two or more abnormal results among the three parasympathetic tests was defined as definite CAN; (4) definite CAN plus orthostatic hypotension was defined as severe CAN; and (5) other combinations, such as early CAN plus orthostatic hypotension or orthostatic hypotension alone, were defined as atypical CAN. For HRV analysis, beat-to-beat heart rate was measured for 5 min at rest. Time-domain analysis was performed based on the standard deviation of normal RR intervals (SDNN) and the root mean square of successive heartbeat interval differences (RMSSD). Frequency-domain analysis was performed using low-frequency (LF: 0.04–0.15 Hz) and high-frequency (HF: 0.15–0.40 Hz) spectral components as well as the LF:HF ratio.

### Definition of CAN recovery

The study outcome was composite events of CAN recovery, which were categorized as partial and complete by comparing baseline and follow-up CARTs. Partial recovery was defined as one-step improvement within the CAN stages (early to normal, definite to early, or severe to definite), or as the disappearance of orthostatic hypotension or one abnormal parasympathetic result in atypical CAN. Complete recovery was defined as the normalization of definite or severe CAN.

### Demographic and clinical assessment

At baseline, demographic data (age, sex, BMI, BP, duration of diabetes, current medications, smoking status, and alcohol-drinking status) was evaluated in the clinic on the same day that the first CARTs were performed. After an 8-h overnight fast, plasma glucose level was measured using the glucose oxidase method; serum C-peptide level was measured in duplicate with immunoradiometric assays (Beckman Coulter, Fullerton, CA, USA), and lipid profiles were assayed using a Hitachi 7600 auto analyzer (Hitachi Instruments Service, Tokyo, Japan). HbA1c level (reference interval: 4.0–6.0%) was measured using high-performance liquid chromatography on a VARIANT II TURBO analyzer (Bio-Rad Laboratories, Hercules, CA, USA). GFR was estimated using Chronic Kidney Disease Epidemiology Collaboration equations [20]. Urinary albumin concentration (μg) was measured using a turbidimetric immunoassay. Urinary creatinine concentration (mg) was measured using a colorimetric method, and the albumin to creatinine ratio (ACR [mg/g]) was calculated by dividing the urinary albumin concentration by the urinary creatinine concentration [21]. Microalbuminuria was defined as an ACR ranging from 30 to 299 mg/g creatine, and macroalbuminuria was defined as an ACR of ≥ 300 mg/g creatinine.

For eye examination, retinal images were obtained using a digital fundus camera (TRC-NW6S; Topcon, Tokyo, Japan) equipped with a Nikon D-80 digital camera (Nikon, Tokyo, Japan), and digital fundus images were obtained from all participants in the same year CARTs were performed. After maximal dilatation of the pupil, a digital retinal image centered on the fovea was obtained for each eye (four images per person in total). If both eyes were rated at different stages, then the grade of the worst eye was used. Both carotid arteries were scanned using a high-resolution B-mode ultrasound machine (VIVID; General Electric, Horten, Norway) and an EKO 7 system (Samsung Medison Co., Ltd., Cypress, CA, USA) equipped with a 7-MHz linear transducer. Carotid intima-media thickness (CIMT) was measured at three points on the far wall of a 1 cm-long section of the common carotid artery in proximity to the carotid bulb. The mean value of six measurements from the right and left carotid arteries was used as the mean CIMT.

On follow-up, BP, body weight, BMI, lipid profiles, fasting plasma glucose (FPG), HbA1c, and alterations in medications (stops or starts) data were collected on the same day as last CARTs were performed. Change represents the value of each variable on follow-up subtracted from the value obtained at baseline. Mean HbA1c was calculated using consecutive data. HbA1c variability was evaluated based on visit to visit standard deviation (SD) and coefficient of variance (CV), with the SD adjusted for the number of measurements (adjusted SD = SD/[n/(n − 1)]^0.5^) because a small number of measurements can lead to overestimation of SD [22].

### Statistical analysis

The clinical characteristics of patients are presented as the mean with SD, median with interquartile range (IQR), or number with percentage. The student’s *t* test and the Mann–Whitney *U* test were used to assess differences in continuous variables between groups, and the Chi square test and McNemar test were used to assess differences in the proportion of categorical variables. Log-transformation was used to achieve a normal distribution in cases of skewed data.

For multivariate logistic regression, two methods were used: only variables that had associations with *P* < 0.10 on univariate logistic regression were selected for inclusion in multivariate logistic regression using the enter method in order to demonstrate the association between each clinical factor and CAN recovery (composite events of partial and complete recovery or complete recovery only). As an additional check given the large number of parameters identified as potentially relevant, a forward–backward stepwise selection process was used as well. No multicollinearity was found when we defined it as a variance inflation factor (VIF) ≥ 5 [23]. The correlation between changes in CAN indices and change in variables was analyzed via linear regression analyses.

To determine the relative importance of clinical factors associated with CAN recovery, we used dominance analysis [24]. The general dominance weight for a variable was calculated from the McFadden R^2^ statistic, and a standardized weight representing the proportional contribution of each variable [24]. Receiver operating characteristic (ROC) analyses were performed to find the optimal age cut-off value for predicting CAN recovery. All statistical analyses were performed with STATA version 14.0 (StataCorp LP, College Station, TX, USA), and a *P* value < 0.05 was considered statistically significant in two-tailed tests.

## Results

### Baseline and follow-up characteristics of study subjects according to CAN recovery

A total of 29.9% (n = 227) of the enrolled subjects exhibited CAN recovery, but only 1.2% (n = 9) recovered completely. Among patients without CAN recovery, those 490 (64.6% of total subjects) showed no change in CAN status and 42 (5.5% of total subjects) experienced CAN progression.

The clinical characteristics of the study subjects are listed in Table 1. At baseline, patients with CAN recovery were younger, more likely to be male, less likely to have other diabetes complications such as micro/macroalbuminuria or increased CIMT, and had a shorter duration of diabetes, higher diastolic BP, and higher TG, C-peptide, and eGFR levels than those who did not experience CAN recovery. The proportion of patients using a statin, angiotensin-converting-enzyme (ACE) inhibitor/angiotensin-converting-enzyme (ARB), calcium channel blocker (CCB), or anti-platelet/anti-coagulant was significantly lower in the group with CAN recovery than in the group without, whereas the proportion of patients using insulin, an oral anti-diabetes drug, or a beta blocker did not differ between the two groups.

**Table 1:** Baseline characteristics according to recovery from cardiovascular autonomic neuropathy in subjects with type 2 diabetes

	Non-recovery (n = 532)	Recovery (n = 227)	*P* value
Age (years)	61.4 ± 7.9	53.3 ± 10.8	< 0.001
Male, n (%)	294 (55.3)	147 (64.8)	0.015
Body weight (kg)	65.8 ± 10.1	68.8 ± 12.7	0.001
Body mass index (kg/m^2^)	24.9 ± 3.0	25.0 ± 3.4	0.783
Any use of alcohol, n (%)	69 (13.0)	39 (17.2)	0.129
Current smoker, n (%)	84 (15.8)	32 (14.1)	0.554
Duration of diabetes (years)	11.5 ± 7.4	8.4 ± 6.2	< 0.001
Systolic BP (mmHg)	127.1 ± 15.7	125.2 ± 15.1	0.127
Diastolic BP (mmHg)	76.2 ± 10.6	78.4 ± 11.7	0.010
Lipid profiles (mg/dL)			
Total cholesterol	157.9 ± 28.8	161.5 ± 31.0	0.125
Triglycerides	123.2 ± 55.1	136.4 ± 75.1	0.007
HDL-C	51.4 ± 13.3	51.4 ± 13.6	0.937
LDL-C	91.2 ± 25.6	93.5 ± 25.8	0.243
Fasting plasma glucose (mg/dL)	144.7 ± 62.2	150.7 ± 52.7	0.204
HbA1c (%)	7.4 ± 4.9	7.4 ± 1.6	0.939
HbA1c (mmol/mol)	57.8 ± 53.2	57.5 ± 17.3	0.939
Fasting C-peptide (ng/ml)^a^	2.2 ± 1.0	2.4 ± 1.2	0.041
Estimated GFR (mL/min/1.73 m^2^)	79.8 ± 16.0	84.5 ± 16.2	< 0.001
Other diabetic complications			
Presence of retinopathy, n (%)^b^	93 (18.2)	27 (12.4)	0.054
Presence of micro/macroalbuminuria, n (%)	96 (18.0)	27 (11.9)	0.035
Mean CIMT (mm)^c^	0.79 ± 0.21	0.71 ± 0.19	< 0.001
Use of insulin, n (%)	53 (10.0)	24 (10.6)	0.799
Use of oral anti-diabetes drug, n (%)	501 (94.2)	210 (92.5)	0.390
Use of statin, n (%)	346 (65.0)	130 (57.3)	0.043
Use of anti-hypertensive drug, n (%)	260 (48.9)	75 (33.0)	< 0.001
ACE inhibitor or ARB	225 (42.3)	66 (29.1)	0.001
Calcium channel blocker	102 (19.2)	23 (10.1)	0.002
Thiazide	83 (15.6)	23 (10.1)	0.109
Beta-blocker	27 (5.1)	10 (4.4)	0.695
Diuretics	83 (15.6)	23 (10.1)	0.028
Use of anti-platelet/anti-coagulant	244 (45.9)	62 (27.3)	< 0.001
Indices of heart rate variability			
Ln SDNN (ms)	3.0 ± 0.5	3.1 ± 0.5	0.061
Ln RMSSD (ms)	2.7 ± 0.6	2.7 ± 0.6	0.137
Ln LF (ms^2^)	4.0 ± 1.2	4.2 ± 1.2	0.097
Ln HF (ms^2^)	3.7 ± 1.2	3.9 ± 1.2	0.052
Ln LF:HF	0.3 ± 1.0	0.4 ± 1.0	0.195
Total CAN score	1.1 ± 0.3	1.1 ± 0.3	0.751

BP, blood pressure; HDL-C, high density lipoprotein cholesterol; LDL-C, low density lipoprotein cholesterol; GFR, glomerular filtration rate; ACR, albumin to creatinine ratio; CIMT, carotid intima-media thickness; ACE, angiotensin converting enzyme; ARB, angiotensin II receptor blockers; SDNN, standard deviation of all NN intervals; RMSSD, root mean square of the successive differences; LF, low frequency; HF, high frequency; CAN, cardiovascular autonomic neuropathy

^a^Measured in 687 subjects

^b^Measured in 727 subjects

^c^Measured in 461 subjects

During 2–3 years of follow-up, HbA1c levels (median number of measurements 7, IQR 6–9) were measured every 3–6 months. There were no differences in FPG, mean HbA1c, or HbA1c variability (adjusted SD and CV), but HbA1c reduction between baseline and follow-up was more prominent in subjects with CAN recovery than in those without. Body weight was significantly decreased in the recovery group, while BMI, BP, and lipid profiles did not change. The proportion of patients who stopped or started insulin, statins, anti-hypertensive medications or anti-coagulants/anti-platelets did not significantly change during the observation period (Table 2). However, oral anti-diabetes drugs were more likely to be altered in subjects with CAN recovery (49.8% vs. 38.5%, *P* = 0.004) than in those without.

**Table 2:** Change in clinical variables and indices of cardiovascular autonomic neuropathy over 2–3 years

Change in variables (Δ)	Non-recovery (n = 532)	Recovery (n = 227)	*P* value
Δ Body weight (kg)	− 0.1 ± 3.3	− 0.7 ± 3.4	0.047
Δ Body mass index (kg/m^2^)	− 0.1 ± 1.2	− 0.2 ± 1.2	0.217
Δ Systolic BP (mmHg)	− 8.4 ± 17.4	− 6.7 ± 18.0	0.218
Δ Diastolic BP (mmHg)	− 1.5 ± 29.7	− 1.6 ± 11.0	0.935
Δ Total cholesterol (mg/dL)	− 6.8 ± 31.6	− 9.9 ± 38.7	0.245
Δ Triglycerides (mg/dL)	5.1 ± 58.4	− 2.7 ± 75.8	0.126
Δ HDL-C (mg/dL)	2.6 ± 24.9	0.9 ± 11.3	0.340
Δ LDL-C (mg/dL)	− 5.9 ± 34.1	− 9.3 ± 33.6	0.212
Δ Fasting plasma glucose (mg/dL)	− 6.7 ± 66.7	− 9.8 ± 58.7	0.540
Change in HbA1c			
ΔHbA1c (%)	0.18 ± 1.18	− 0.26 ± 1.23	< 0.001
Mean HbA1c (%)	7.1 ± 1.0	7.1 ± 0.9	0.850
Adjusted SD of HbA1c (%)^a^	0.50 ± 0.45	0.48 ± 0.40	0.521
CV of HbA1c	6.7 ± 5.8	6.4 ± 4.7	0.397
Change in medication			
Δ Use of insulin, n (%)	24 (4.5)	13 (5.7)	0.477
Δ Use of oral anti-diabetes drug, n (%)	205 (38.5)	113 (49.8)	0.004
Δ Use of statin, n (%)	96 (18.0)	41 (18.1)	0.996
Δ Use of anti-hypertensive drug, n (%)	133 (25.0)	47 (20.8)	0.214
Δ Use of anti-platelet/anti-coagulant, n (%)	88 (16.5)	27 (11.9)	0.107
Change in indices of heart rate variability			
Δ Ln SDNN (ms)	− 0.02 ± 0.52	0.15 ± 0.45	< 0.001
Δ Ln RMSSD (ms)	− 0.03 ± 0.64	0.02 ± 0.59	0.302
Δ Ln LF (ms^2^)	0.33 ± 1.04	0.40 ± 1.07	0.341
Δ Ln HF (ms^2^)	− 0.04 ± 1.22	0.04 ± 1.06	0.384
Δ Ln LF:HF	0.08 ± 1.23	− 0.18 ± 1.23	0.009
Δ Total CAN score	0.1 ± 0.4	− 1.1 ± 0.2	< 0.001

ΔChange represents a variable’s value at follow-up subtracted from the value obtained at baseline

BP, blood pressure; HDL-C, high density lipoprotein cholesterol; LDL-C, low density lipoprotein cholesterol; SDNN, standard deviation of all NN intervals; RMSSD, root mean square of the successive differences; LF, low frequency; HF, high frequency; CAN, cardiovascular autonomic neuropathy

^a^SD of HbA1c divided by [n/(n–1)]^0.5^, where n is the number of HbA1c measurements

Among various HRV indices, log-transformed SDNN was significantly increased and log-transformed LF:HF ratio was significantly decreased during follow-up in subjects with CAN recovery, while none of the HRV indices differed between two groups at baseline (Tables 1 and 2). A significant decrease in total CAN score was also observed in subjects with CAN recovery (Table 2).

### Clinical characteristics according to each category of CAN recovery

**Fig. 2:**
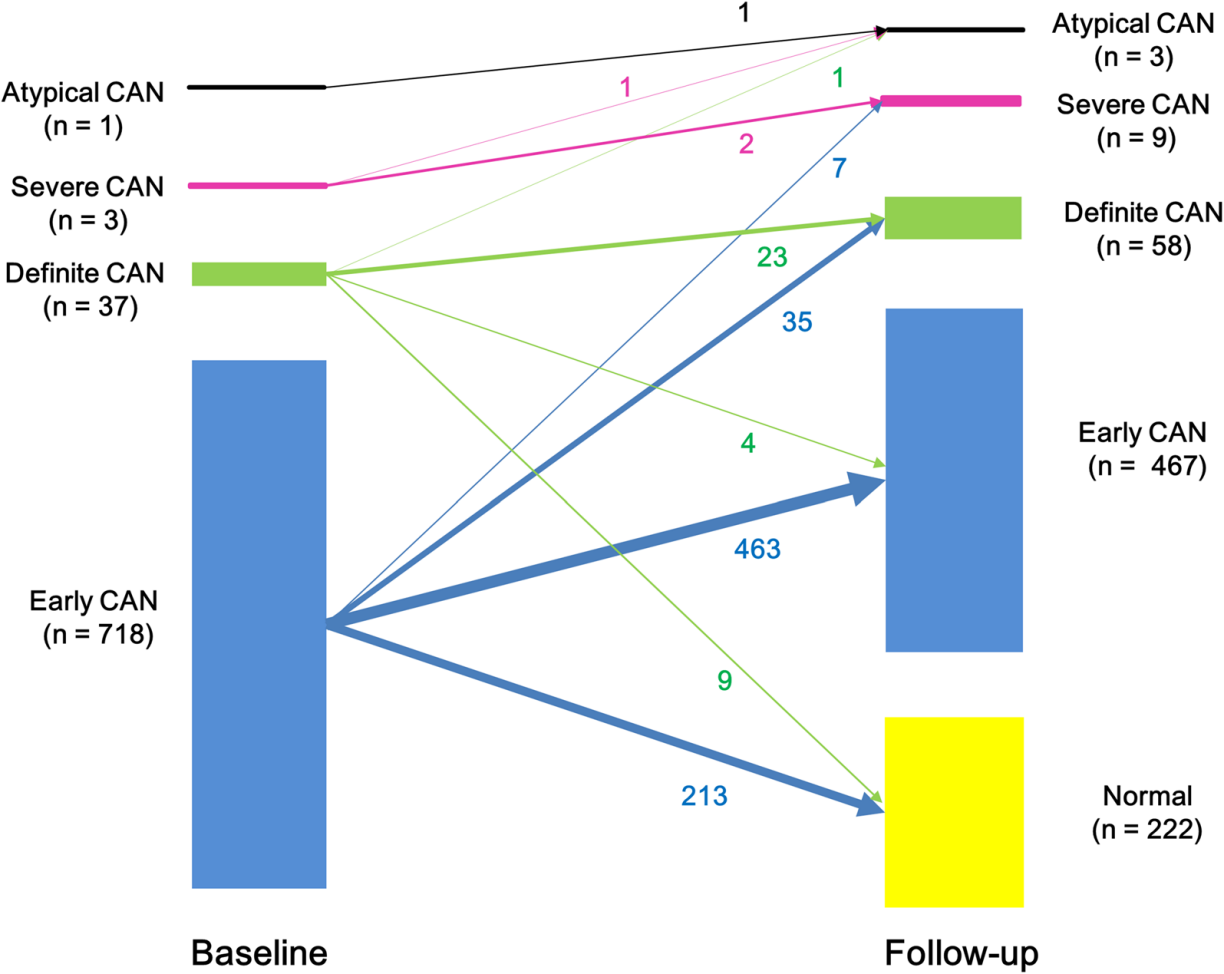
Change in each category of cardiovascular autonomic neuropathy over 2–3 years

CAN recovery was categorized into three groups: early to normal, definite or severe to early, and definite or severe to normal (complete recovery). Change from the early stage to normal status accounted for the majority of CAN recovery (n = 213, 93.8%), and the second highest proportion was recovery from definite or severe CAN to normal (n = 9, 4.0%; Fig. 2). The subjects who recovered from definite or severe CAN to normal status were the youngest (Additional file 1: Table S2). The duration of diabetes was shorter and serum TG level was lower in the group that recovered from definite or severe CAN to normal status compared to the non-recovery group. Baseline HbA1c, HbA1c change, and mean HbA1c were also the lowest in patients who recovered completely, while SD and CV of HbA1c did not vary among the four groups (Additional file 1: Table S2).

### Clinical correlates for composite events of CAN recovery

In univariate logistic regression analyses, baseline values such as younger age, male sex, lower body weight, shorter duration of diabetes, higher diastolic BP, higher TG level, higher eGFR, lower C-peptide level, no micro/macroalbuminuria, smaller mean CIMT, no use of statins, no use of CCBs or ACE inhibitors/ARBs, and no use of an anti-platelets/anti-coagulants were significantly associated with composite events of CAN recovery. Body weight reduction and alterations in oral anti-diabetes drugs were also associated with composite CAN recovery (Table 3). Among glycemic parameters, only HbA1c reduction was a significant factor in composite CAN recovery, while baseline HbA1c, baseline FPG, mean HbA1c, adjusted SD of HbA1c, and CV of HbA1c were not correlated with composite recovery in univariate analysis.

**Table 3:** Significant associated factors for composite recovery of cardiovascular autonomic neuropathy in subjects with type 2 diabetes

	Partial + complete recovery (n = 227)					
	Univariate OR (95% CI)	*P* value	Adjusted OR (95% CI)^a^	*P* value	Adjusted OR (95% CI)^b^	*P* value
Baseline variables						
Younger age (per 5-year decrease)	1.62 (1.47–1.78)	< 0.001	1.49 (1.25–1.78)	< 0.001	1.52 (1.33–1.75)	< 0.001
Male	1.49 (1.08–2.05)	0.015	1.65 (0.87–3.13)	0.124		
Body weight (per 1 kg decrease)	1.03 (1.01–1.04)	0.001	1.02 (0.99–1.05)	0.185		
Duration of diabetes (per 5 year decrease)	1.41 (1.24–1.59)	< 0.001	1.33 (1.05–1.67)	0.016	1.37 (1.13–1.67)	0.002
Diastolic BP (per 10 mmHg decrease)	0.83 (0.72–0.96)	0.010	0.94 (0.73–1.21)	0.631		
Triglycerides (per 10 mg/dL decrease)	0.97 (0.94–0.99)	0.008	0.99 (0.95–1.03)	0.690		
Fasting C-peptide (per 1 ng/mL increase)^c^	0.86 (1.74–0.99)	0.043	1.25 (0.96–1.62)	0.092		
Estimated GFR (per 10 mL/min/1.73 m^2^ increase)	1.20 (1.09–1.33)	< 0.001	1.08 (0.91–1.28)	0.396		
Presence of retinopathy, yes^d^	0.64 (0.40–1.01)	0.056	1.35 (0.68–2.68)	0.390		
Presence of micro/macroalbuminuria, yes	0.61 (0.39–0.97)	0.037	0.34 (0.15–0.78)	0.011	0.34 (0.16–0.75)	0.007
Mean CIMT (per 1 mm decrese)^e^	10.12 (2.87–35.69)	< 0.001	2.39 (0.56–10.19)	0.238		
Use of statin, yes	0.72 (0.52–0.99)	0.043	1.04 (0.61–1.78)	0.890		
Use of CCB or ACE inhibitor/ARB, yes	0.52 (0.37–0.71)	< 0.001	0.88 (0.50–1.53)	0.643		
Use of anti-platelet/anti-coagulant, yes	0.44 (0.32–0.62)	< 0.001	0.88 (0.50–1.55)	0.659		
Change in variables						
Δ body weight (per 1 kg decrease)	1.05 (1.01–1.10)	0.048	1.11 (1.02–1.21)	0.016	1.10 (1.02–1.19)	0.015
Δ HbA1c (per 1% decrease)	1.36 (1.19–1.56)	< 0.001	1.32 (1.05–1.67)	0.019	1.35 (1.08–1.67)	0.007
Δ Change in oral anti-diabetes drug, yes	1.60 (1.17–2.18)	0.004	0.81 (0.47–1.42)	0.465		

^a^Only factors with* P* < 0.10 on univariate logistic regression were presented and included in the multivariate logistic regression

^b^Odd ratios were analyzed by a forward–backward stepwise selection process

^c^Measured in 687 subjects

^d^Measured in 727 subjects

^e^Measured in 461 subjects

After adjustment for variables with *P* < 0.10 on univariate logistic regression analyses, younger age (OR per 5-year decrease 1.49; 95% confidence interval [CI] 1.27–1.78, *P* < 0.001), shorter duration of diabetes (OR per 5-year decrease 1.33; 95% CI 1.05–1.67, *P* = 0.016), body weight reduction (OR per 1 kg decrease 1.11; 95% CI 1.02–1.21, *P* = 0.016), and HbA1c reduction (OR per 1% decrease 1.32; 95% CI 1.05–1.67, *P* = 0.019) remained significant factors in composite CAN recovery. These factors remained significant after stepwise selection in multivariate logistic regression analysis.

We considered age, sex, duration of diabetes, change in body weight, change in HbA1c and the degree of albuminuria as categorical variables (Additional file 2: Figure S1). In analysis, 10-year age categories demonstrated a clear decreasing tendency in CAN recovery with age, as shown in Additional file 2: Figure S1a: 88.5% at age < 40 years, 63.7% at age 40–49 years, 30.6% at age 50–59 years, 18.6% at age 60–69 years, and 15.5% at age ≥ 70 years (*P* for trend < 0.001). Male patients had a higher rate of CAN recovery than female patients (Additional file 2: Figure S1b; 33.3% vs. 25.2%, *P* = 0.025). Shorter duration of diabetes (Additional file 2: Figure S1c; 45.6% with a duration of < 5 years, 33.4% with a duration of 5–9 years, 24.7% with a duration of 10–19 years, and 13.2% with a duration of ≥ 20 years; *P* for trend < 0.001), decreased body weight (Additional file 2: Figure S1d; 34.9% with weight change < -5.0%, 28.7% with weight change − 5.0 to 5.0%, and 26.1% with weight change > 5.0%; *P* for trend = 0.027), and tertiles of HbA1c change (Additional file 2: Figure S1e; 37.3% with HbA1c change < − 0.2%, 29.0% with HbA1 change − 0.2 to 0.3%, and 23.9% with HbA1c change > 0.3%; *P* for trend = 0.002) were also associated with an increase in composite CAN recovery. However, there was no significant correlation between composite CAN recovery and the degree of albuminuria (Additional file 2: Figure S1f).

### Clinical correlates for the complete CAN recovery

In univariate analyses (Table 4), younger age, change in oral anti-diabetes drugs, and change in anti-hypertensive drugs were significantly associated with complete CAN recovery. In multivariate logistic regression analysis (Table 4), only younger age (OR per 5-year decrease 1.63; 95% CI 1.07–2.49, *P* = 0.023) was significantly associated with complete CAN recovery.

**Table 4:** Significant associated factors for the complete recovery of cardiovascular autonomic neuropathy in subjects with type 2 diabetes

	Complete recovery (n = 9)					
	Univariate OR (95% CI)	*P* value	Adjusted OR (95% CI)^a^	*P* value	Adjusted OR (95% CI)^b^	*P* value
Baseline variables						
Younger age (per 5-year decrease)	1.67 (1.26–2.20)	< 0.001	1.63 (1.07–2.49)	0.023	1.67 (1.26–2.23)	< 0.001
Total cholesterol (per 10 mg/dL decrease)	1.27 (0.97–1.67)	0.088	1.24 (0.96–1.61)	0.099		
HbA1c (per 1% decrease)	0.97 (0.94–1.00)	0.065	1.54 (0.78–3.05)	0.212		
Change in variables						
Δ Change in oral anti-diabetes drug, yes	4.94 (1.02–23.94)	0.047	3.44 (0.65–18.07)	0.145		
Δ Change in anti-hypertensive drug, yes	0.10 (0.02–0.52)	0.006	0.96 (0.84–10.91)	0.971		

^a^Only factors with *P* < 0.10 on univariate logistic regression were presented and included in the multivariate logistic regression

^b^Odd ratios were analyzed by a forward–backward stepwise selection process

### The relative significance of factors associated with CAN recovery

We used dominance analysis to compare the relative importance of age, duration of diabetes, presence of micro/macroalbuminuria, body weight reduction, and HbA1c reduction, which were significantly associated with CAN recovery in multivariate logistic models (Additional file 1: Table S2). Younger age had the highest value for explaining the association with CAN recovery, HbA1c reduction the second highest, shorter duration of diabetes the third highest, body weight reduction the second lowest, and no presence of micro/macroalbuminuria the lowest. Because age is the most significant factor for CAN recovery, we performed ROC analysis to determine the optimal cut-off value for age. The threshold was 58.4 years with 63.5% sensitivity and 65.6% specificity.

### The association of change in HRV indices and total CAN score with change in clinical variables

Table 5 demonstrated the linear relationship of change in HRV indices and total CAN score with change in variables. Log-transformed SDNN was inversely correlated with change in HbA1c, and log-transformed RMSSD was inversely correlated with change in TG level. Female sex was inversely correlated with change in LF, but changes in clinical variables were not associated with HF. Age, duration of diabetes, change in total cholesterol, and alterations in anti-hypertensive medications were all positively correlated with increase in LF: HF ratio over time. Increase in total CAN score was positively associated with age, duration of diabetes, body weight gain, increase in HbA1c, mean HbA1c, adjusted SD of HbA1c, or CV of HbA1c, alterations in anti-hypertensive drugs, and alterations in anti-platelets/anti-coagulants.

**Table 5:** Linear associations between change in heart rate variability indices and change in clinical variables

	Standardized β					
	Δ Ln SDNN	Δ Ln RMSSD	Δ LF	Δ HF	Δ LF: HF	Δ Total CAN score
Age (years)	− 0.070	0.021	− 0.330	0.003	0.073*	0.266***
Sex (female)	− 0.050	0.023	− 0.173***	0.030	0.002	0.064
Duration of diabetes (year)	− 0.057	− 0.033	0.040	− 0.032	0.086*	0.161***
Current smoking	0.038	0.038	0.025	0.020	0.030	0.051
Δ Body weight (kg)	− 0.028	− 0.020	− 0.004	− 0.041	0.015	0.074*
Δ BMI (kg/m^2^)	− 0.030	− 0.024	− 0.002	− 0.041	0.022	0.049
Δ SBP (mmHg)	− 0.013	0.008	− 0.017	− 0.004	0.031	− 0.012
Δ DBP (mmHg	0.013	0.001	0.001	0.007	0.049	0.004
Δ Total cholesterol (mg/dL)	− 0.012	− 0.020	0.010	− 0.026	0.077*	0.002
Δ Triglycerides (mg/dL)	− 0.040	− 0.080*	0.049	− 0.072	0.055	0.054
Δ HDL-C (mg/dL)	0.017	0.015	0.008	0.037	− 0.046	0.010
Δ LDL-C (mg/dL)	0.015	0.002	0.033	0.026	0.046	0.014
Δ Fasting plasma glucose (mg/dL)	− 0.060	− 0.006	0.026	− 0.012	0.019	0.004
Δ HbA1c (%)	− 0.079*	− 0.044	− 0.020	− 0.043	0.045	0.248***
Mean HbA1c (%)	− 0.062	− 0.070	0.036	− 0.056	0.032	0.085*
Adjusted SD of HbA1c (%)	− 0.004	− 0.040	0.060	− 0.030	− 0.022	0.083*
CV of HbA1c	0.011	− 0.029	0.053	0.011	− 0.040	0.081*
Δ Change in insulin, yes	− 0.009	− 0.002	− 0.015	0.019	0.071	0.016
Δ Change in oral anti-diabetes drug, yes	0.019	− 0.020	0.025	− 0.013	− 0.029	− 0.060
Δ Change in statin, yes	0.032	0.012	0.030	0.015	0.001	− 0.023
Δ Change in anti-hypertensive drug, yes	− 0.030	− 0.031	0.066	− 0.025	0.097**	0.175***
Δ Change in anti-platelet/anti-coagulant, yes	0.046	0.062	− 0.015	0.048	0.003	0.076*

* *P* < 0.05, ** *P* < 0.01, and *** *P* < 0.001

## Discussion

To the best of our knowledge, this study is the first to investigate the reversibility of CAN and associated factors in patients with type 2 diabetes. Age is the most important factor for CAN recovery, including recovery from definite or severe CAN stages; CAN recovery increased 49% for every 5-year decrease in patient age. Age had stronger positive correlations with change in total CAN score than change in glycemic parameters, and also had positive correlations with change in LF:HF ratio, which estimates the ratio between sympathetic and parasympathetic nervous activity. A shorter duration of diabetes, absence of albuminuria, body weight reduction, and HbA1c reduction were other factors which were significantly associated with CAN recovery.

### CAN reversibility

Partial recovery of CAN accounted for 96.0% of composite recovery, and change from early CAN to normal accounted for the majority (97.7%). Considering the fact that interventions such as ACE inhibitor use [25], favorable glycemic control [14], exercise [26], or body weight loss [27] improved CAN from the early stage, but not from more advanced stages, the higher proportion of early CAN in this study might have led to overestimation of recovery. In addition, less of our participants were female (41.9%) than male (58.1%), which is associated with CAN progression [27]. Included participants also had relatively well-controlled diabetes with baseline HbA1c of 7.3 ± 1.4%, change in HbA1c of 0.04 ± 1.21% during follow-up, and mean HbA1c of 7.1 ± 0.1%, compared to those who were included in other studies for CAN progression [28]. Recovery from definite- or severe CAN to normal status was only observed in younger patients regardless of conventional CAN-associated factors such as glycemic control or duration of diabetes. This suggests that CAN recovery in older patients might be more difficult, as most age-related damage to the autonomic nerves is considered irreversible.

### Factors associated with CAN recovery

Although the subjects and CAN definition included in previous studies have been diverse, glycemic status is undoubtedly important in the incidence and progression of diabetic CAN. Both the time and frequency domains of the HRV indices in the early stage of CAN were improved by intensive glycemic control in a few patients with type 1 diabetes who had poorly controlled HbA1c (mean 9.0%) and CAN improved when HbA1c level improved to approximately 1.0% decrease over the course of 12 months [14]. Intensive insulin therapy providing strict glycemic control improved the median nerve conduction velocity, postural hypotension, and CV of the R-R intervals in patients with type 2 diabetes, whereas conventionally treated patients showed significant deterioration in autonomic function [29]. However, the contribution of glycemic control to CAN was less strong in type 2 diabetes than in type 1 diabetes. Multifactorial treatment targeting hypertension and hyperlipidemia using lifestyle modifications (diet, smoking cessation, and physical exercise) has successfully reduced the risk of CAN in patients with type 2 diabetes, but not in those with type 1 diabetes [4, 26–28]. This supports findings in our dominance analysis that younger age is the most significant factor for CAN recovery, more than HbA1c reduction in particular.

Weight loss induced by graded and supervised exercise for 12 weeks improved HRV in middle aged, insulin-dependent diabetes patients with early-stage CAN when their glycemic control was satisfactory [26]. The effect of body weight reduction on CAN recovery was also not superior to that of HbA1c reduction in this study. Since diabetic complications share common mechanisms, microvascular complications such as retinopathy and albuminuria predict CAN progression [30]; in addition, subclinical atherosclerosis represented by CIMT and carotid plaque is associated with CAN in patients with type 2 diabetes [31]. Because there is little evidence that control of other diabetic complications could lead to CAN recovery or vice versa, it is only speculation that the co-existence of other diabetic complications might affect CAN recovery based on our data. The non-recovery group had a higher proportion of albuminuria and lower eGFR in addition to higher CIMT compared to the recovery group, despite a higher proportion of statin, ACE inhibitor/ARB or CCB, and anti-platelet/anti-coagulant use. Because of the protective effects of statin [32], ACE inhibitor/ARB [25, 33], or CCB [34] use on the modulation of cardiac autonomic function through the reduction of oxidative stress, the higher proportion of statin, ACE inhibitor/ARB, or CCB users in the non-recovery group might reflect increased risk of CVD.

### The effect of aging and diabetes on CAN pathophysiology

Age is generally considered to be one of the most important factors in autonomic nerve function [35] because aging itself can reduce the autonomic responsiveness of HRV [36]. An increase in cardiovascular sympathetic activity and a decrease in cardiac parasympathetic activity were found in response to age-related reductions in baroreceptor sensitivity [37] and increases in circulating levels of norepinephrine at an advanced age [38]. Thus, the blunted vagal modulation of the heart could be related to a progressive decline in sinoatrial conduction and sinus node recovery time with age [39]. Other studies have revealed an increase in empty Schwann cell bands and a reduced number of fibers in the vagus nerve in healthy old people [38]. Age‐related sarcopenia also contributes to the loss of innervating motor neurons and the denervation of muscle fibers even in hearts [40], with leading cardiac dysfunction [41].

Diabetes further exacerbates the normal senescence of the cardiovascular system with age [42]. Driven by hyperglycemia, the formation of advanced glycation end products (AGEs) is accelerated in diabetes, and the interaction of AGEs with endothelial and other cells accumulating in atherosclerotic plaques provides a mechanism that can augment vascular dysfunction [43]. Due to reduced blood flow to synapses and neurons, AGEs cause synaptic inactivity and neuronal death [44]. However, unlike the aging process for autonomic dysfunction, worsening of the nerves can be either minimized or reversed by appropriate intervention, especially if CAN is detected early [45]. Although the adjusted SD and CV of HbA1c had a modest positive correlation with change in total CAN score (but not the incidence of CAN recovery), a rapid reduction in HbA1c over 2–3 years was strongly associated with both a decrease in total CAN score and the incidence of CAN recovery in this study. Compared to chronic hyperglycemia, glycemic variability is associated with greater reactive oxygen species production and vascular damage, which are both pathophysiologic causes of CAN [46].

### Limitations

This study has several limitations. First, our results are drawn from the patients enrolled at one general hospital, so they are not representative of all type 2 diabetes patients in Korea. Second, selection bias might have occurred because we intentionally excluded subjects with CVD, which could affect the results of CARTs. Third, the number of complete recovery events was small (1.2% of total patients), which can cause logistic regression analyses to sharply underestimate probability. Fourth, we cannot completely avoid the influence of medication that affect autonomic function. Another limitation is that our data contained no information about patient diet, exercise, or smoking cessation, all of which can affect autonomic function. Fifth, echocardiographic indices and other electrocardiogram parameters were not available for analysis in this study. Lastly, whether reversing CAN will translate directly into cardiac protection remains unclear. However, we expect that the beneficial effect of CAN recovery on CVD development could be extrapolated based on recent findings that progressing CAN (defined as parasympathetic nerve dysfunction) is associated with an increased risk of CVD in patients with type 2 diabetes [28]. This study had an observational design, and randomized controlled trials (RCTs) are still needed to confirm the causality of the observed associations with CAN recovery.

## Conclusion

Younger age is the most important factor for CAN recovery in patients with type 2 diabetes, including recovery from the definite or severe stage. Other factors such as HbA1c reduction, body weight reduction, the absence of concurrent micro/macroalbuminuria, and shorter duration of diabetes are also significantly associated with CAN recovery. Therefore, careful monitoring for CAN in relatively young patients with type 2 diabetes and intensive glycemic control with lifestyle modifications could facilitate rapid CAN recovery. Further evidence is needed to clarify the causality of the associations among these factors and CAN recovery, as well as the optimal duration of CAN monitoring.

## Additional files

**Additional file 1: Table S1.** Reference values of the four autonomic function tests and the severity scores expressed as points. **Table S2.** Descriptive characteristics according to the recovery status of cardiovascular autonomic neuropathy. **Table S3.** Relative importance of the clinical factors associated with composite events of cardiovascular autonomic neuropathy recovery.

**Additional file 2: Figure S1.** Proportion of cardiovascular autonomic neuropathy (CAN) recovery according to the categorical strata of each variable. The black bar represents complete recovery of CAN, and the grey bar represents partial recovery of CAN. A. According to age category (years): younger than 40, 40–49, 50–59, 60–69, and 70 and older. B. According to sex: male and female. C. According to diabetes duration category (years): 0–4, 5–9, 10–19, and 20 and more. D. According to body weight change categories: less than -5.0, -5.0 to 5.0, and over 5.0%. E. According to change in HbA1c tertiles: less than -0.2 (lowest), -0.2–3.0 (middle) and over 3.0% (highest). F. According to degree of albuminuria: normal, microalbuminuria, and macroalbuminuria.

## Abbreviations

ACE: angiotensin-converting enzyme
ACR: albumin to creatinine ratio
AGEs: advanced glycation end products
ARB: angiotensin II receptor blocker
BMI: body mass index
BP: blood pressure
CAN: cardiovascular autonomic neuropathy
CARTs: cardiovascular autonomic reflex tests
CCB: calcium channel blocker
CIMT: carotid intima-media thickness
CV: coefficient of variance
CVD: cardiovascular disease
eGFR: estimated glomerular filtration rate
HF: high frequency
FPG: fasting plasma glucose
HRV: heart rate variability
IQR: interquartile range
LF: low frequency
RMSSD: root mean square of successive heartbeat interval differences
ROC: receiver operating characteristic
SD: standard deviation
SDNN: standard deviation of normal RR
TG: triglycerides
