# Supplementary material for: Clinical factors associated with the recovery of cardiovascular autonomic neuropathy in patients with type 2 diabetes mellitus
Source: Cardiovasc Diabetol. 2019 Mar 11;18:29. doi: 10.1186/s12933-019-0830-4 (PMC6410519; doi:10.1186/s12933-019-0830-4)
Supplement: Supplementary file 1 — Additional file 1: Table S1. Reference values of the four autonomic function tests and the severity scores expressed as points. Table S2. Descriptive characteristics according to the recovery status of cardiovascular autonomic neuropathy. Table S3. Relative importance of the clinical factors associated with composite events of cardiovascular autonomic neuropathy recovery. [file 12933_2019_830_MOESM1_ESM.docx]

**Additional Table S1**. Reference values of the five autonomic function tests and the severity scores expressed as points.

| Autonomic function test | Measurement | Defined values | | |
| --- | --- | --- | --- | --- |
|  |  | Normal(0) | Borderline(0.5) | Abnormal(1) |
| ΔHR to deep breathing | E:I ratio | ≥ 1.06^a^ |  | < 1.06^a^ |
| Lying to standing ΔHR | 30 : 15 ratio | ≥ 1.07^b^ |  | < 1.07^b^ |
| Valsalva maneuver | Valsalva ratio | ≥ 1.12^c^ |  | < 1.12^c^ |
| Postural BP change | Fall in systolic BP | ≤ 10 | 11-29 | ≥ 30 |

ΔHR, heart rate change; BP (mm Hg), blood pressure; E:I ratio, expiration to inspiration ratio.

^a^Lower limit of the age-specific reference range of E:I ratio : age 20-24 years, 1.17; age 25-29, 1.15; age 30-34, 1.13; age 35-39, 1.12; age 40-44, 1.10; age 45-49, 1.08; age 50-54, 1.07; age 55-59, 1.06; age 60-64, 1.04; age 65-69, 1.03; and age 70-75, 1.02.

^b^Lower limit of the age-specific reference range of 30:15 ratio : age 20-24 years 1.15; age 25-29, 1.14; age 30-34, 1.12; age 35-39, 1.11; age 40-44, 1.10; age 45-49, 1.09; age 50-54, 1.08; age 55-59, 1.07; age 60-64, 1.07; age 65-69, 1.06; and age 70-75, 1.06.

^c^Lower limit of the age-specific reference range of valsalva ratio : age 20-24 years, 1.43; age 25-29, 1.38; age 30-34, 1.33; age 35-39, 1.28; age 40-44, 1.24; age 45-49, 1.20; age 50-54, 1.16; age 55-59, 1.12; age 60-64, 1.08; age 65-69, 1.04; and age 70-75, 1.00.

**Additional Table S2.** Descriptive characteristics according to the recovery status of cardiovascular autonomic neuropathy

|  | **No recovery** | **Early**  **🡪 normal** | **Definite to severe**  **🡪 early** | **Definite to severe**  **🡪 normal** | ***P* value** |
| --- | --- | --- | --- | --- | --- |
| **Incidence, n (%)** | 532 (70.2) | 213 (28.1) | 4 (0.5) | 9 (1.2) |  |
| **Levels at baseline** |  |  |  |  |  |
| Age (years) | 61.3 ± 7.9 | 53.8 ± 10.6 | 49.5 ±11.4 | 41.6 ± 11.5 | <0.001 |
| Male, n (%) | 294 (55.3) | 140 (65.7) | 2 (50.0) | 5 (55.6) | 0.049 |
| Body weight (kg) | 65.8 ± 10.1 | 68.7 ± 12.9 | 69.4 ± 10.0 | 66.9 ± 12.8 | 0.008 |
| Body mass index (kg/m^2^) | 24.9 ± 3.0 | 25.0 ± 3.4 | 24.0 ± 3.5 | 24.9 ± 3.7 | 0.929 |
| Any use of alcohol, n (%) | 69 (13.0) | 39 (18.3) | 0 (0.0) | 0 (0.0) | 0.166 |
| Current smoker, n (%) | 84 (15.8) | 30 (14.1) | 1 (25.0) | 1 (11.1) | 0.588 |
| Duration of diabetes (years) | 11.5 ± 7.4 | 8.4 ± 6.2 | 8.9 ± 6.0 | 7.3 ± 5.1 | <0.001 |
| Systolic BP (mmHg) | 127.1 ± 15.7 | 125.4 ± 14.7 | 121.2 ± 23.2 | 114.5 ± 11.2 | 0.168 |
| Diastolic BP (mmHg) | 76.2 ± 10.6 | 78.6 ± 11.4 | 75.3 ± 4.2 | 75.4 ± 19.3 | 0.053 |
| Lipid profiles (mg/dL) |  |  |  |  |  |
| Total cholesterol | 157.9 ± 28.8 | 162.2 ± 31.2 | 170.5 ± 38.5 | 141.1 ± 15.4 | 0.067 |
| Triglycerides | 123.0 ± 55.1 | 136.0 ± 75.4 | 199.8 ± 82.6 | 109.7 ± 51.0 | 0.005 |
| HDL-C | 51.5 ± 13.3 | 51.5 ± 13.2 | 51.3 ± 22.0 | 48.9 ± 19.4 | 0.952 |
| LDL-C | 91.1 ± 25.6 | 94.1 ± 26.0 | 100.8 ± 23.3 | 77.4 ± 16.4 | 0.144 |
| Fasting plasma glucose (mg/dL) | 144.7 ± 62.2 | 149.7 ± 48.2 | 157.0 ± 56.3 | 130.9 ± 37.0 | 0.001 |
| HbA1c (%) | 7.2 ± 1.4 | 7.3 ± 1.4 | 7.6 ± 1.6 | 6.9 ± 0.6 | <0.001 |
| Fasting C-peptide (ng/ml)^b^ | 2.2 ± 1.0 | 2.4 ± 1.2 | 1.9 ± 1.0 | 2.8 ± 1.1 | 0.122 |
| Estimated GFR (mL/min/1.73m^2^) | 79.8 ± 16.0 | 84.5 ± 15.6 | 101.7 ± 22.6 | 81.1 ± 23.2 | <0.001 |
| Other diabetic complications |  |  |  |  |  |
| Presence of retinopathy, n (%) | 93 (18.2) | 25 (12.3) | 1 (25.0) | 1 (11.1) | 0.092 |
| Presence of micro/macroalbuminuria, n (%) | 96 (18.0) | 24 (11.3) | 1 (25.0) | 2 (22.2) | 0.140 |
| Mean CIMT (mm) | 0.79 ± 0.21 | 0.71 ± 0.18 | 0.62 ± 0.02 | 0.61 ± 0.08 | 0.001 |
| Use of insulin, n (%) | 53 (10.0) | 20 (9.4) | 2 (50.0) | 2 (22.2) | 0.293 |
| Use of oral anti-diabetes drug, n (%) | 501 (94.2) | 196 (92.0) | 4 (100.0) | 9 (100.0) | 0.725 |
| Use of statin, n (%) | 346 (65.0) | 121 (56.8) | 3 (75.0) | 5 (55.6) | 0.067 |
| Use of anti-hypertensive drug, n (%) | 260 (48.9) | 70 (32.9) | 0 (0.0) | 5 (1.5) | <0.001 |
| Use of anti-platelet/anti-coagulant, n (%) | 244 (45.9) | 58 (27.2) | 0 (0.0) | 3 (1.0) | <0.001 |
| **Change in variables over 2-3 years** |  |  |  |  |  |
| Δ Body weight (kg) | -0.14 ± 3.34 | -0.65 ± 3.35 | -0.44 ± 4.50 | -0.77 ± 3.24 | 0.303 |
| Δ Body mass index (kg/m^2^) | -0.08 ± 1.24 | -0.19 ± 1.14 | -0.12 ± 1.67 | -0.34 ± 1.26 | 0.707 |
| Δ Systolic BP (mmHg) | -8.4 ± 17.4 | -6.5 ± 18.1 | -11.0 ± 18.7 | -10.1 ± 17.3 | 0.552 |
| Δ Diastolic BP (mmHg) | -1.5 ± 29.7 | -1.8 ± 10.8 | -0.1 ± 14.0 | -3.3 ± 13.5 | 0.996 |
| Δ Total cholesterol (mg/dL) | -6.8 ± 31.6 | -11.5 ± 38.7 | 22.0 ± 14.0 | 12.4 ± 36.7 | 0.024 |
| Δ Triglycerides (mg/dL) | 5.1 ± 58.4 | -3.5 ± 75.0 | -3.0 ± 15.6 | 2.5 ± 54.4 | 0.183 |
| Δ HDL-C (mg/dL) | 2.6 ± 24.9 | 1.1 ± 10.5 | 0.8 ± 7.8 | -4.1 ± 24.1 | 0.234 |
| Δ LDL-C (mg/dL) | -5.9 ± 34.1 | -10.3 ± 33.2 | 7.0 ± 31.7 | 4.7 ± 44.4 | 0.704 |
| Δ Fasting plasma glucose (mg/dL) | -6.7 ± 66.7 | -11.2 ± 54.1 | 3.7 ± 44.1 | 25.5 ± 21.48 | 0.552 |
| Δ HbA1c (%) | 0.18 ± 1.18 | -0.24 ± 1.23 | -0.28 ± 0.98 | -1.23 ± 1.92 | <0.001 |
| Mean HbA1c (%) | 7.1 ± 1.0 | 7.1 ± 0.9 | 7.9 ± 1.5 | 6.9 ± 0.8 | <0.001 |
| Adjusted SD of HbA1c (%) | 0.50 ± 0.45 | 0.47 ± 0.40 | 0.99 ± 0.08 | 0.46 ± 0.38 | 0.108 |
| CV of HbA1c | 6.7 ± 5.8 | 6.3 ± 4.7 | 10.5 ± 2.0 | 6.2 ± 4.8 | 0.404 |
| Δ Use of insulin, n (%) | 24 (4.5) | 13 (6.1) | 0 (0.0) | 0 (0.0) | 0.522 |
| Δ Use of oral anti-diabetes drug, n (%) | 205 (38.5) | 104 (48.8) | 2 (50.0) | 7 (77.8) | 0.001 |
| Δ Use of statin, n (%) | 96 (18.0) | 40 (18.8) | 0 (0.0) | 1 (0.7) | 0.754 |
| Δ Use of anti-hypertensive drug, n (%) | 133 (25.0) | 46 (21.6) | 0 (0.0) | 1 (0.6) | 0.125 |
| Δ Use of anti-platelet/anti-coagulant, n (%) | 88 (16.5) | 27 (12.7) | 0 (0.0) | 0 (0.0) | 0.050 |
| Follow-up duration (years) | 2.8 ± 0.7 | 2.8 ± 0.7 | 2.7 ± 0.6 | 2.9 ± 0.6 | 0.923 |

Δ Change represents a variable’s value at follow-up subtracted from the value obtained at baseline.

**Additional Table S3.** Relative importance of the clinical factors associated with the composite events of cardiovascular autonomic neuropathy recovery

|  | **Standardized weight^a^** | **Rank** |
| --- | --- | --- |
| Younger age | 0.6999 | 1 |
| HbA1c reduction | 0.1328 | 2 |
| Shorter duration of diabetes | 0.1108 | 3 |
| Body weight reduction | 0.0316 | 4 |
| No presence of micro/macroalbuminuria | 0.0249 | 5 |

^a^Standardized weight is the general dominance weight from McFadden R^2^ normed or standardized to be out of 100% [19].
