# Supplementary material for: Clinical factors associated with the recovery of cardiovascular autonomic neuropathy in patients with type 2 diabetes mellitus
Source: Cardiovasc Diabetol. 2019 Mar 11;18:29. doi: 10.1186/s12933-019-0830-4 (PMC6410519; doi:10.1186/s12933-019-0830-4)
Supplement: Supplementary file 2 — Additional file 2: Figure S1. Proportion of cardiovascular autonomic neuropathy (CAN) recovery according to the categorical strata of each variable. The black bar represents complete recovery of CAN, and the grey bar represents partial recovery of CAN. A. According to age category (years): younger than 40, 40–49, 50–59, 60–69, and 70 and older. B. According to sex: male and female. C. According to diabetes duration category (years): 0–4, 5–9, 10–19, and 20 and more. D. According to body weight change categories: less than -5.0, -5.0 to 5.0, and over 5.0%. E. According to change in HbA1c tertiles: less than -0.2 (lowest), -0.2–3.0 (middle) and over 3.0% (highest). F. According to degree of albuminuria: normal, microalbuminuria, and macroalbuminuria. [file 12933_2019_830_MOESM2_ESM.pdf]

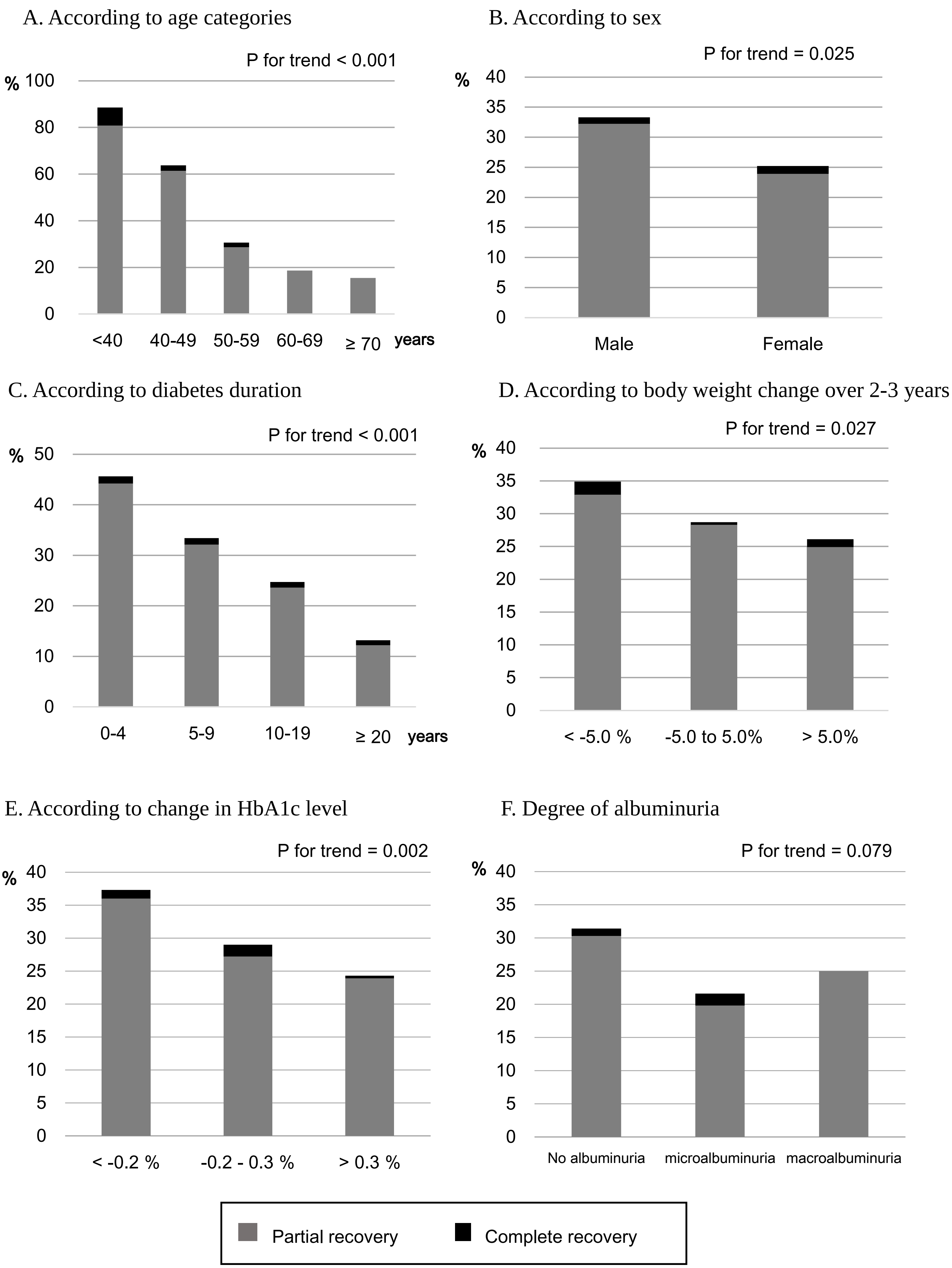
A. According to age categories
P for trend < 0.001
%
<40
40-49
50-59
60-69
≥ 70
years
B. According to sex
P for trend = 0.025
%
Male
Female
C. According to diabetes duration
P for trend < 0.001
%
0-4
5-9
10-19
≥ 20
years
D. According to body weight change over 2-3 years
P for trend = 0.027
%
< -5.0 %
-5.0 to 5.0%
> 5.0%
E. According to change in HbA1c level
P for trend = 0.002
%
< -0.2 %
-0.2 - 0.3 %
> 0.3 %
F. Degree of albuminuria
P for trend = 0.079
%
No albuminuria
microalbuminuria
macroalbuminuria
Partial recovery
Complete recovery
